# Thicket and Mesh: How the Outer Membrane Can Resist Tension Imposed by the Cell Wall

**DOI:** 10.1021/acs.jpcb.3c08510

**Published:** 2024-05-24

**Authors:** David Ryoo, Hyea Hwang, James C. Gumbart

**Affiliations:** † Interdisciplinary Bioengineering Graduate Program, Georgia Institute of Technology, Atlanta, Georgia 30332, United States; ‡ School of Materials Science and Engineering, Georgia Institute of Technology, Atlanta, Georgia 30332, United States; § School of Physics, Georgia Institute of Technology, Atlanta, Georgia 30332, United States

## Abstract

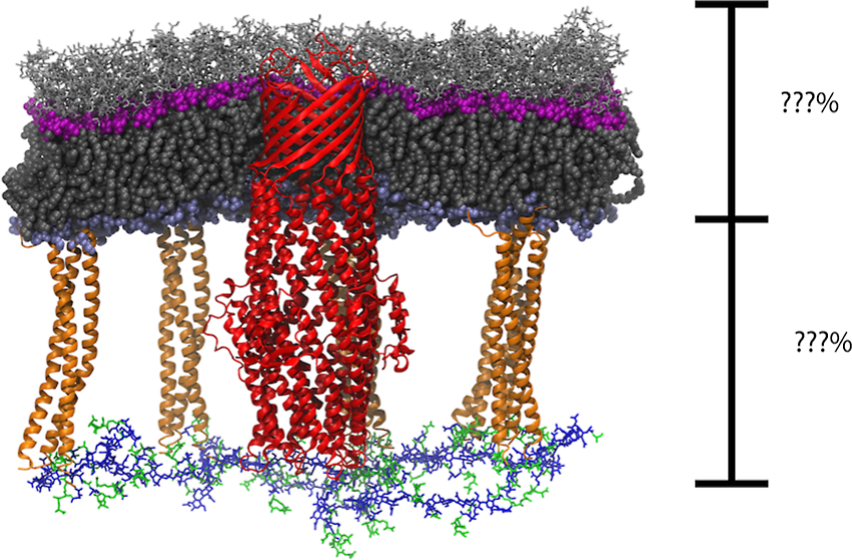

The cell envelope of Gram-negative bacteria is composed of an outer membrane (OM) and an inner membrane (IM) and a peptidoglycan cell wall (CW) between them. Combined with Braun’s lipoprotein (Lpp), which connects the OM and the CW, and numerous membrane proteins that exist in both OM and IM, the cell envelope creates a mechanically stable environment that resists various physical and chemical perturbations to the cell, including turgor pressure caused by the solute concentration difference between the cytoplasm of the cell and the extracellular environment. Previous computational studies have explored how individual components (OM, IM, and CW) can resist turgor pressure although combinations of them have been less well studied. To that end, we constructed multiple OM-CW systems, including the Lpp connections with the CW under increasing degrees of strain. The results show that the OM can effectively resist the tension imposed by the CW, shrinking by only 3–5% in area even when the CW is stretched to 2.5× its relaxed area. The area expansion modulus of the system increases with increasing CW strain, although the OM remains a significant contributor to the envelope’s mechanical stability. Additionally, we find that when the protein TolC is embedded in the OM, its stiffness increases.

## Introduction

The bacterial cell envelope provides physical and chemical protection from the extracellular elements.^1,2^ For Gram-negative bacteria, the cell envelope is composed of a two-membrane system: an asymmetric outer membrane (OM) and a symmetric inner membrane (IM). The asymmetric OM is composed of lipopolysaccharides (LPSs) in the outer leaflet and phospholipids in the inner leaflet, while the symmetric IM is composed of phospholipids in both leaflets. LPS comprises lipid A, core oligosaccharides, and O-antigens^3^ and can be considered as a thicket, acting as a permeability barrier.^4^ In the space between OM and IM (called the periplasm), there is a mesh-like peptidoglycan network known as the cell wall (CW).^5,6^ Unlike Gram-positive bacteria, which only have one membrane and a thick, external CW,^7,8^ the CW of Gram-negative bacteria is a single-layered mesh composed of chains of repeated *N*-acetyl glucosamine—*N*-acetyl muramic acid disaccharides cross-linked by short oligopeptides.^9^ The three components that make up the cell envelope collectively create a mechanically stable and selectively permeable barrier that protects bacteria from harmful small molecules, such as antimicrobial agents.^2,10^

The mechanical stability of Gram-negative bacteria has been a topic of interest over the years,^10−13^ as it has been implicated in various functions of the bacteria including cell growth,^13^ pathogenesis^10^ (such as adhesins^12^ and pili^14^), and even macroscale interactions^15,16^ (including biofilm formation^17^). Of the different forces that Gram-negative bacteria have to endure, their resistance to turgor pressure has been studied extensively.^11,13,18−21^ Turgor pressure comes from the difference in solute concentrations between the extracellular environment of the bacteria and the cytoplasmic environment, and the proper regulation of such pressure was implicated in bacterial cell growth.^13^ Estimates of the turgor pressure experienced by Gram-negative bacteria range from 0.3 to 5 atm (0.03–0.5 MPa).^22−25^ How different components of the cell envelope contribute to resistance to turgor pressure is only recently beginning to be resolved.

Hwang et al. evaluated the turgor pressure resistance of the three components of the cell envelope individually using both computational and experimental techniques.^19^ The results indicated that the IM and OM experience stress softening behavior under increasing surface tension, while the CW displays strain stiffening instead. Combining the contributions from each component, it was shown that the OM and CW share the burden at low values of the turgor pressure, but the CW dominates the response at sufficiently high values.^19^ This conclusion gives more context to the finding that OM plays an essential role in load-bearing,^18^ yet also supports the classical notion that the CW is the dominant element,^26^ at least in extreme circumstances. Furthermore, in *Escherichia coli* and related bacteria, the OM and CW are covalently linked by Braun’s lipoprotein (Lpp),^21,27,28^ which contributes to the stiffness of the combined system as well.^18,20,29^

In the present work, to account for the combination of OM, CW, and Lpp, we have constructed and simulated multiple OM and CW systems linked by Lpp. In particular, we initialized the CW under different degrees of strain to quantify the competition between it and the OM. In addition, we have constructed an OM-CW system with the outer-membrane protein (OMP) TolC. TolC is a part of the multidrug efflux pump, AcrAB-TolC,^30,31^ and in recent studies, it was found that TolC interacts with the CW as well as the OM.^32−34^ Our results show that the OM can withstand tensions imposed by CW, compressing only marginally in response. Additionally, we find that the presence of TolC increases the overall stiffness of the cell envelope, in agreement with a recent study finding that the MacAB-TolC complex contributes to membrane rigidity.^35^

## Methods

### System Generation

The *E. coli* CW model used here is the avg17 model from Gumbart et al.,^6^ which has an average glycan strand length of 17 disaccharides and a cross-linking fraction of ∼0.5, meaning approximately half of the peptides are linked and half are free (see Table 1 for the dimensions of the CW). It was originally in a relaxed state (no applied surface tension), which was used to construct the CW 1.0× system. It was then stretched by applying multiple, increasing uniform surface tensions in the *xy* plane for 10 ns each until it reached twice and 2.5 times its original area, which were used to construct the CW 2.0× and CW 2.5× systems, respectively.^19^ We note that even at 2.5 times the original area, the peptide bonds in the CW are not unphysiologically stretched (Figure S1). The TolC-containing CW-OM system was also taken from a previous study.^33^

**Table 1:** CW Dimensions of the OM-CW Systems

system	width (Å)	length (Å)	lateral area (Å^2^)
1.0×	78.1	154.9	12,101
2.0×	125.3	191.9	24,041
2.5×	147.8	203.0	30,008
TolC	191.8	191.8	36,785

Four different OM systems were generated using CHARMM-GUI.^36,37^ For each, the outer leaflet of the OM was composed of LPS from the *E. coli* K12 strain, which has no O-antigen.^19,38^ The inner leaflet of the OM was built using a ratio of 75:20:5 for 1-palmitoyl(16:0)-2-palmitoleoyl(16:1 *cis*-9)-phosphatidylethanolamine (PPPE), 1-palmitoyl(16:0)-2-vacenoyl(18:1 *cis*-11)-phosphatidylglycerol (PVPG), and 1,1′-palmitoyl-2,2′-vacenoyl cardiolipin (net charge of −2e) (PVCL2), respectively.^38^ The number of lipids for both inner and outer leaflets was determined from the CW area. Based on the area per lipid for LPS^39^ (178 Å^2^) and that for inner leaflet lipids on average^40^ (64 Å^2^), we calculated the number of LPS in the outer leaflet and the total number of lipids in the inner leaflet. Then, we calculated the number of PPPE, PVPG, and PVCL2 based on the inner-leaflet composition mentioned above. The total number of lipids/LPS for inner and outer leaflets used for each system is shown in Table S1. LPS was neutralized using divalent ions with magnesium (Mg^2+^) for lipid A and calcium (Ca^2+^) for the LPS core sugars based on prior studies identifying these ions in their respective regions^41,42^ (see Table S2 for numbers of magnesium and calcium ions). The system was solvated and ionized to a concentration of 0.15 M NaCl.

Four, seven, nine, and nine copies of Lpp trimers (PDB ID: 1EQ7(28)) were covalently bonded to the CW 1.0×, CW 2.0×, CW 2.5×, and TolC CWs, respectively. The area density of Lpp was estimated to be 36–38 nm^2^ per trimer.^1,19,33^ Each Lpp was lipidated at the N-terminus with tripalmitoyl-S-glyceryl-cysteine residues, and one from each trimer^43^ at the C-terminus was covalently bonded to the uncross-linked meso-diaminopimelate (mesoDAP) residue of the CW after removing the two following alanine residues in the CW peptide chain.^44^

The OM systems were modified to match the lateral dimensions of the CW by moving the lipids, water, and ions of the CHARMM-GUI outputs using visual molecular dynamics (VMD).^45^ Then, we ran each of the OM systems for 1.5 μs to assess the equilibrated area in comparison to the desired CW area. The OM areas averaged over the last 500 ns came within 0.3, 0.5, and 1.0% for the CW 1.0×, CW 2.0×, and CW 2.5× areas, respectively. Next, the OM system was positioned above the CW and Lpp system, and the lipidated N-terminus of each Lpp was embedded within the hydrophobic core of the OM system. As the lipidated N-terminus of each Lpp monomer accounts for 1.5 lipids within the OM (three acyl chains), 1–2 inner-leaflet lipids near each lipidated N-terminus in the system were removed. For example, as there are 12 lipidated N-termini for the CW 1.0× system (four Lpp trimers), 18 inner leaflet lipids close to the N-termini were removed. The system was later solvated and re-ionized in order to maintain the bulk concentration of 0.15 M NaCl.

To quantify the degree to which the tension imposed by the strained CWs affects the OM, we also created “CW damaged” versions for all four systems. In the original systems, the CW is bonded to itself across the periodic boundaries, effectively making it infinite. In the damaged versions, the periodic bonds were broken, making the CW a finite patch. For analysis of the area change over time of the combined systems, the reference point was taken to be the average of the CW area and the equilibrated OM area, hereafter referred to as the normalized area.

### Molecular Dynamics

All-atom molecular dynamics (MD) simulations were performed using NAMD3^46^ along with the CHARMM36m force field for proteins,^47,48^ the CHARMM36 force field for lipids,^49^ and TIP3P water.^50^ All simulations were performed under periodic boundary conditions with a cutoff at 12 Å for short-range electrostatic and Lennard-Jones interactions and a force-based switching function starting at 10 Å for the latter. The particle-mesh Ewald method^51^ with a grid spacing of less than 1 Å was used for the calculation of long-range electrostatic interactions. Bonds between a heavy atom and a hydrogen atom were maintained to be rigid, while all other bonds remained flexible. A time step of 4 fs was used, enabled by the application of hydrogen mass repartitioning.^52,53^

Four OM systems were equilibrated for 1.5 μs before being combined with the CW and Lpp. Each full OM-CW system (with OM, Lpp, and CW) was held at a constant area for 100 ns, followed by equilibration for 2 μs with two replicas (using two different random seeds) under an isothermal–isobaric ensemble (NPT) at 310 K and 1 bar. A Langevin thermostat with a damping coefficient of 1 ps^–1^ was used for temperature control, and a Langevin piston with a period of 0.1 ps and decay of 0.05 ps was used for pressure control. For the first replica of the CW 1.0× system, the equilibrium simulation was extended to 3.4 μs to observe the stability of the area over a longer timescale (Figure S2). For the CW-damaged systems described above, after breaking the covalent bonds across the periodic boundaries, each system was minimized for 1000 steps and then equilibrated for 2 μs in two replicas with the same conditions as the OM-CW systems.

For each of the two replicas, after 2 μs of equilibration, lateral surface tension was applied to the system for 200 ns per surface tension target for a series of increasing values. The surface tension γ(*t*) in the system can be directly measured from the difference between lateral and normal pressures as follows:
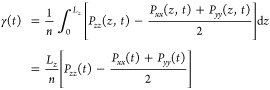
1where *L*_*z*_ is the length of the system in the *Z* direction, *P*_*xx*_, *P*_*yy*_, and *P*_*zz*_ are the pressures along the *X*-, *Y*-, and *Z*-axes, respectively, and *n* is the number of surfaces (assumed to be two) .^54^ For the pressure along the *Z*-axis, we applied a constant pressure of 1 atm. The surface tension targets applied along the *XY* direction ranged from 0 to 80 dyn/cm in 10 dyn/cm increments (a total of nine different surface tension targets). These surface tension targets were applied consecutively starting from 0 dyn/cm; the actual surface tension was measured in the simulation according to eq 1. The total simulation time was 66 μs. System setup, visualization, and analysis were performed using VMD.

## Results

### OM Is Minimally Compressed by a Strained CW

**Figure 1:**
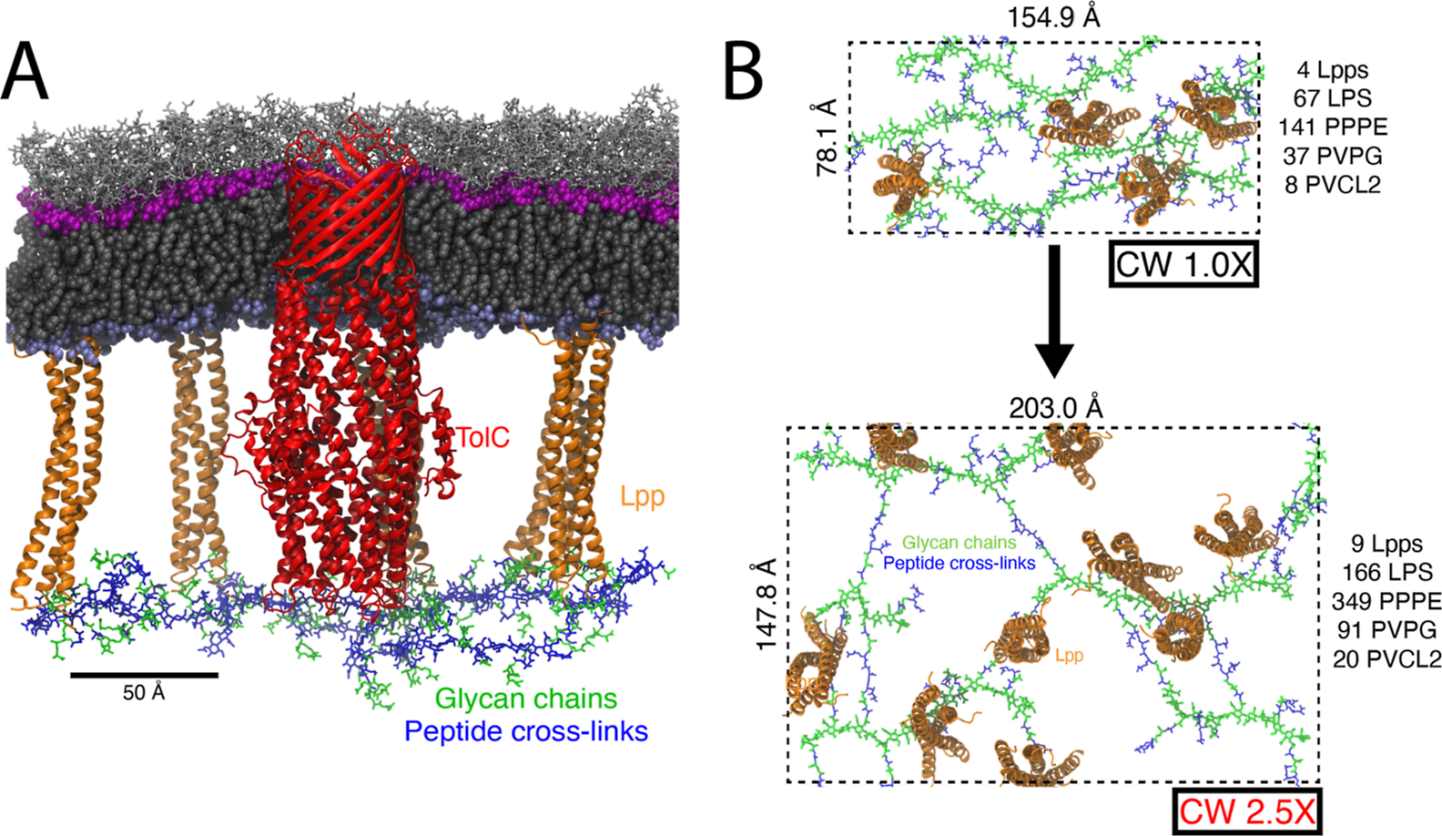
Simulated OM-CW systems. (A) Side view of the OM-CW system of TolC. The sugars in LPS are shown in silver licorice representation and the polar heads of LPS and phospholipids are shown in magenta and ice blue spheres, respectively. The lipid tails for both are shown in gray spheres. The Lpps are shown in orange cartoon representation. For the CW, the glycan chains are shown in green licorice and the peptide cross-links are shown in blue licorice. (B) CW strain. The size of the CW 1.0× system (top) and the CW 2.5× system (bottom) is shown. The number of Lpps, LPS, and lipids used is shown on the right side of the system. Additional labels for Lpps and CW are shown within the CW 2.5× system.

In order to test if the OM along with Lpp responds to the surface tension imposed by the CW, we have constructed three different systems based on the initial CW areas. Specifically, we have built complete OM-CW systems (see Figure 1A) with a relaxed CW (CW 1.0× system; see Figure 1B, top), a CW that has been stretched to twice its relaxed area (CW 2.0× system), and a CW that has been stretched to 2.5 times its relaxed area (CW 2.5× system; see Figure 1B, bottom). For each system, the OM was constructed separately to match the CW’s area, i.e., it was not under strain initially (see Methods). We note that in the living cell, the CW is under extreme tension, while the OM is not.^18^ In addition, we constructed an OM-CW system with TolC embedded in the OM and with a minimally strained (1.32×) CW (TolC system). The details of the systems can be found in the Methods section and in Tables 1, S1, and S2. Each system was held at a constant area for 100 ns followed by constant pressure of 1 atm for 2 μs, run in duplicate.

**Figure 2:**
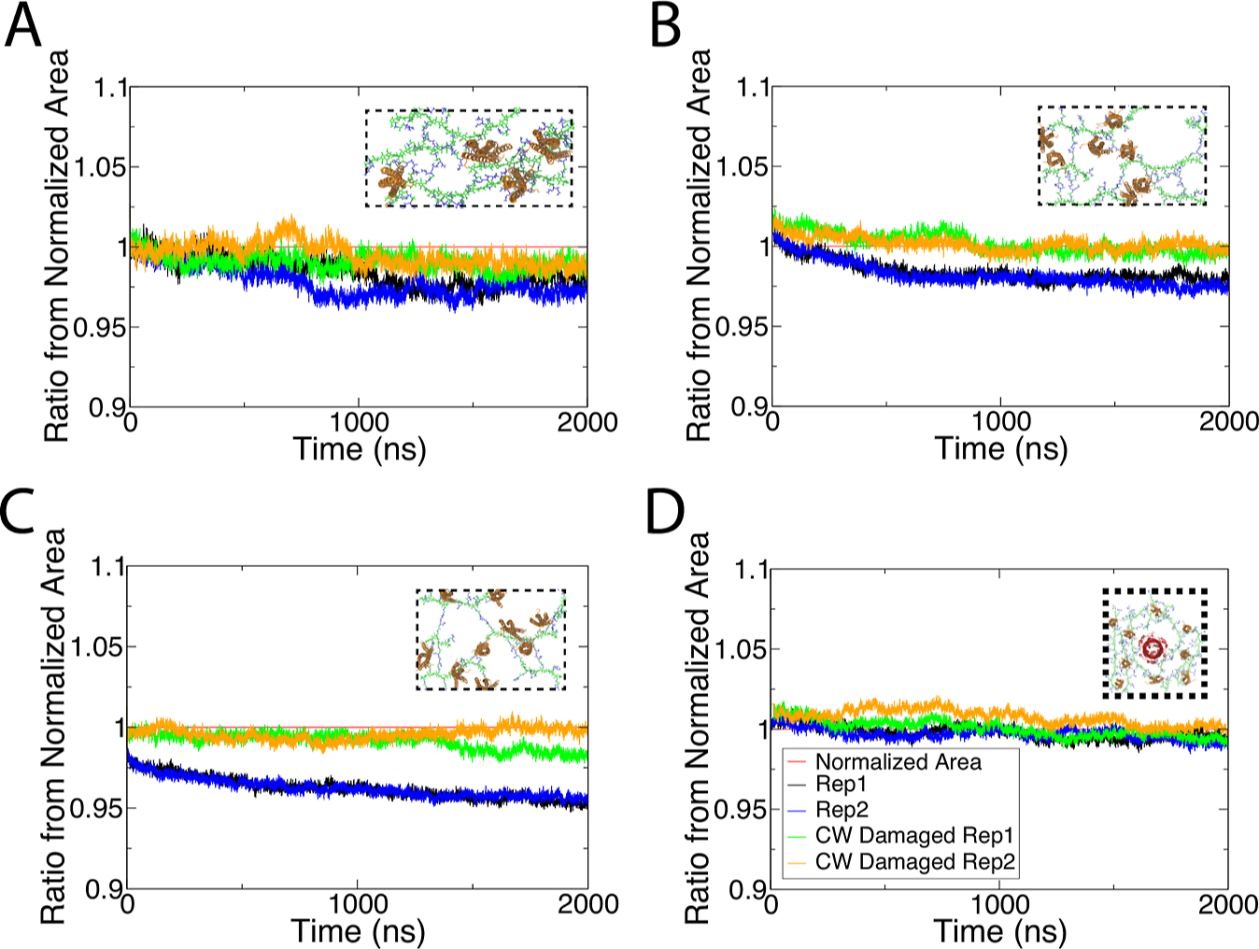
OM withstands tension imposed by the CW. The ratio of membrane area change from the normalized area over time is shown for the (A) CW 1.0× system, (B) CW 2.0× system, (C) CW 2.5× system, and (D) TolC system. The initial CW area is shown as a horizontal red line. Replicas 1 and 2 of each intact-CW system are shown in black and blue, respectively. The replicas with the damaged CW are shown in green and orange, respectively. For each plot, a graphical representation of the CW with Lpp is shown in the inset.

The change in area of all systems over time is shown in Figure 2 (black and blue curves). The area, calculated using the XY dimensions of the simulation box, proved to be stable over time for most systems. To quantify this, we measured the average area of all the systems using the last 200 ns of their respective simulations (Table S3). The CW 1.0× shrank by 1–3%, although extending one replica to 3.4 μs reversed the compression slightly, indicative of long-time-scale fluctuations (Figure S2). Compression was more pronounced and uniform for both replicas of the CW 2.0× and CW 2.5× systems, which shrank by nearly 3 and 5%, respectively. Therefore, while the OM compresses in response to the strained CW, the effect is relatively small compared to the degree of expansion of the CW. The TolC system showed almost no area change over time (Figure 2D), unsurprising given that there was little tension on the CW (1.32×).^33^ In addition, the area change was more stable over time than that for the CW 1.0× system, suggesting that the presence of TolC (or other OMPs) may stiffen the OM, as also found previously.^35,55^

To ensure that the area change over time was not a consequence of how the OM was constructed for each system, we also repeated the simulations but with the CW “damaged”, i.e., the covalent bonds connecting it across the periodic boundaries were broken. In this way, the CW could shrink irrespective of the system’s periodic dimensions. As seen in Figure 2 (orange and green curves), the systems’ areas changed by, at most, 1–2% (Table S4). When comparing to the undamaged-CW systems, the CW 1.0× and TolC systems show negligible differences in areas (Figure 2A,D). The CW 2.0× and CW 2.5× damaged and undamaged systems, on the other hand, show statistically significant differences in areas (Tables S3 and S4), emphasizing the compression induced by an intact CW.

We also considered the possibility that area contraction would alter the physical properties of the OM. We first looked for buckling, curvature, or other distortions; however, the systems appear similar to one another irrespective of CW damage (Figure S3). We also measured the lipid order parameters for all OM components (Figures S4–S6). We see a general, although not uniform, trend toward a modest increase in order with increasing compression imposed by the CW, which is eliminated when the CW is damaged. The OM does not undergo a phase transition in any of the systems.

### Both the CW and OMPs Stiffen the Cell Envelope

In order to determine the mechanical properties of each system, we applied lateral surface tension to all systems with an undamaged CW (see Methods). By applying surface tension and measuring the resulting area expansion, the area expansion modulus, *K*_A_, of the system can be calculated as follows
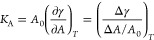
2where *A* is the system area, *A*_0_ is the equilibrium area, *T* is the temperature, and γ is the applied surface tension. This relationship demonstrates that the area expansion modulus is the slope of the applied surface tension vs the area expansion line. Increasing values of surface tension were applied consecutively. Each surface tension was applied for 200 ns, and the last 100 ns of the simulation was used for analysis. Unsurprisingly, imposed surface tension caused the lipid order parameters to decrease (Figures S8–S10).

**Figure 3:**
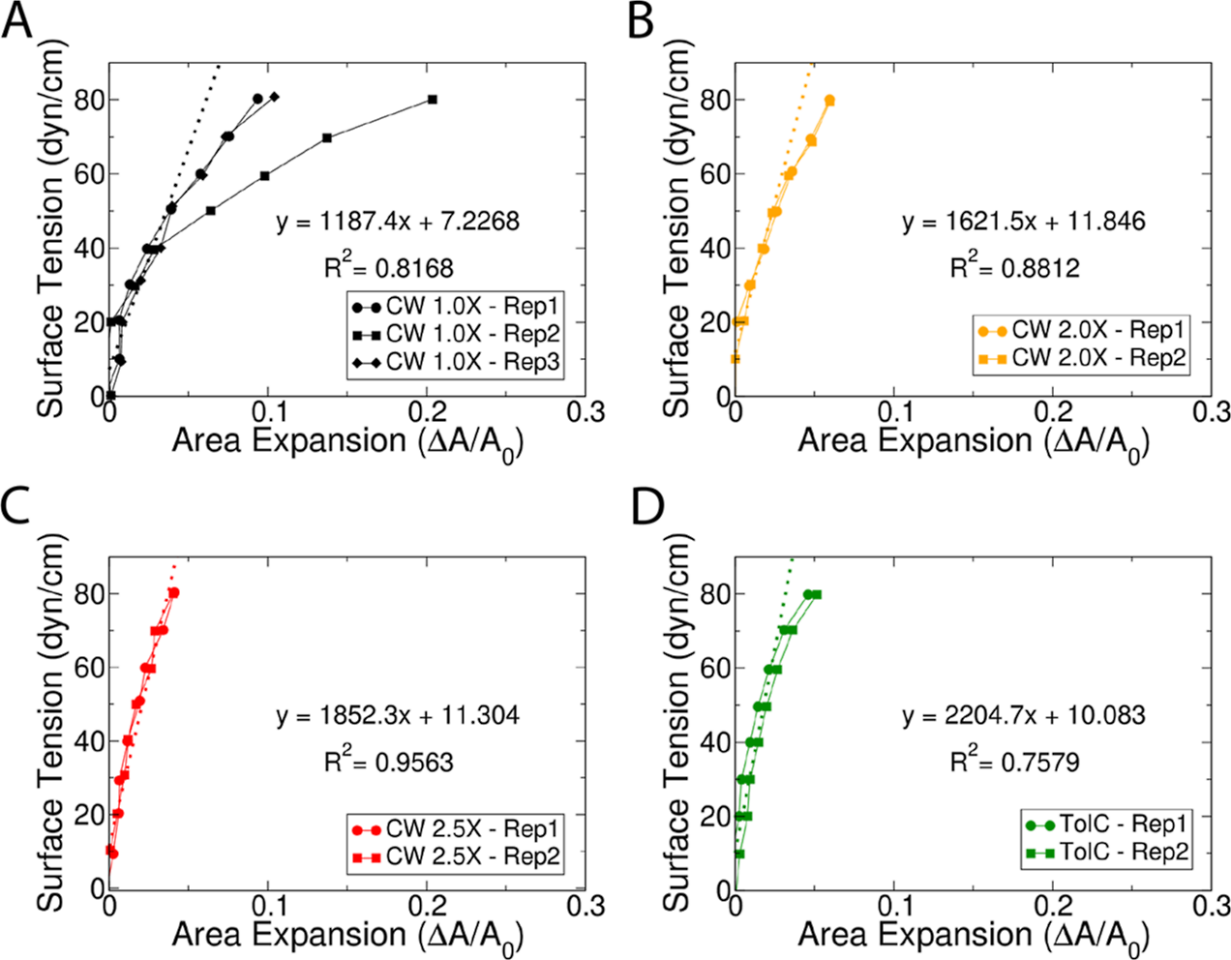
Applied surface tension vs area expansion for the (A) CW 1.0× system (black), (B) CW 2.0× system (orange), (C) CW 2.5× system (red), and (D) TolC system (green). The first replica’s data points are shown as circles, the second replica’s data points are shown as squares, and the third replica's data points are shown as diamonds, if applicable. The linear regressions are shown as dotted lines. The linear regression equation and the R^2^ values for each plots are shown in the middle of the graph.

Plots for all simulations are shown in Figure 3. *K*_A_ was calculated based on the linear region of the applied surface tension vs area expansion plots. The linear region was determined by consecutively performing linear regression for an increasing number of sequential data points starting from 0 dyn/cm. For most systems, including data up to 40 dyn/cm was optimal, after which the *R*^2^ of the fit diminished with the inclusion of additional data points. The second replica of the CW 1.0× system exhibited stress softening, in which *K*_A_ decreased sharply at tensions above ∼40 dyn/cm (see Figure 3A). The trajectory revealed that the OM of this system ruptured at an intermediate tension despite being identical to the first replica (see Figure S7), resulting in the observed stress softening. Thus, a third replica was run for the CW 1.0× system. As for others, this replica was first equilibrated for 2 μs prior to applying surface tension (see area over time in Figure S2). *K*_A_ for the CW 1.0× system was calculated from replicas 1 and 3.

The *K*_A_ values were found to be 1187 dyn/cm for the CW 1.0× system, 1622 dyn/cm for the CW 2.0× system, 1852 dyn/cm for the CW 2.5× system, and 2205 dyn/cm for the TolC system. All values are notably higher than that found in simulations of the OM alone previously (524 dyn/cm).^19^*K*_A_ increased with increasing CW strain, indicative of strain stiffening.^19,25^ For the TolC system, the *K*_A_ was the highest calculated from all the systems tested, even though the CW was initially in a relaxed state. The results suggest that the presence of an OMP (and perhaps especially a cell-envelope-spanning one such as TolC) can stiffen the cell envelope, as also seen previously.^55^ This is in contrast to previous simulations of the OM with OmpF embedded for which no change in *K*_A_ was found.^19^ However, it is notable that OmpF forms a trimer in the OM, and, thus, its interprotein interactions may be softer than protein–lipid interactions for TolC.

## Discussion

In this study, we have examined the collective response of OM-CW systems under different CW strains (CW 1.0×, CW 2.0×, and CW 2.5×), along with a TolC system that contained the OMP TolC with a relaxed CW. We first examined the area change over time for all systems, finding that they contracted only slightly (3–5%) as the OM resisted tension imposed by the strained CW. We also applied increasing surface tensions consecutively from 0 to 80 dyn/cm and calculated the area expansion modulus *K*_A_ (Figure 3). We found *K*_A_ values ranged from 1187 dyn/cm (for the CW 1.0× system) to 1852 dyn/cm for the CW 2.5× system, reflecting an increasing stiffness as a function of the strain on the CW. This strain stiffening was also observed previously for the CW in both simulations^19^ and experiments.^25^

In a previous study, simulations were used to measure the area expansion moduli for the OM and CW separately.^19^ There, *K*_A_ for the OM was determined to be 524 dyn/cm, while for the CW, *K*_A_ depended on the strain, ranging from ∼10 dyn/cm for the relaxed CW up to over 1000 dyn/cm for the 2.5×-strained CW. Combining these suggests an expected *K*_A_ of 535 dyn/cm for the CW 1.0× system, 775 dyn/cm for the CW 2.0× system, and 1575 dyn/cm for the CW 2.5× system, all lower than what we measured here by 300–800 dyn/cm albeit with a similar upward trend. However, we note that Lpp, which covalently links OM and CW together and was included here, also plays a role in stiffening the cell envelope,^18,20,29^ and its contribution may not be purely additive as it affects both the OM and CW.^18^

The OM has become recognized as a major contributor to the bacterial cell’s mechanical stability under the load imposed by turgor pressure.^18,19^ Here, we also find similar contributions from the OM and the CW to the envelope’s area expansion modulus. The OM’s ability to bear a large mechanical load is further emphasized by its minimal compression under tension, even that imposed by a 2.5×-strained CW (Figure 2C). Finally, the contribution of OMPs needs to be considered, including ones that span the OM-CW gap such as TolC, which increased *K*_A_ here (Figure 3D). OMPs’ density and nonuniform distribution in the OM^56^ likely also have an effect on its mechanical properties, which remains to be elucidated.

## Supporting Information Available

Additional system images, plots of order parameters and other additional analysis, and system properties and measurements (PDF)
