# Supplementary material for: Thicket and Mesh: How the Outer Membrane Can Resist Tension Imposed by the Cell Wall
Source: J Phys Chem B. 2024 May 24;128(22):5371–7. doi: 10.1021/acs.jpcb.3c08510 (PMC11163421; doi:10.1021/acs.jpcb.3c08510)
Supplement: Supplementary file 1 — jp3c08510_si_001.pdf [file jp3c08510_si_001.pdf]

Supporting Information:

Thicket-and-Mesh: How the Outer Membrane Can Resist Tension Imposed by the Cell Wall

David Ryoo,[†] Hyea Hwang,[‡] and James C. Gumbart[*,¶]

[†]*Interdisciplinary Bioengineering Graduate Program, Georgia Institute of Technology, Atlanta, GA 30332, USA*

[‡]*School of Materials Science and Engineering, Georgia Institute of Technology, Atlanta, GA 30332, USA*

[¶]*School of Physics, Georgia Institute of Technology, Atlanta, GA 30332, USA*

E-mail: gumbart@physics.gatech.edu

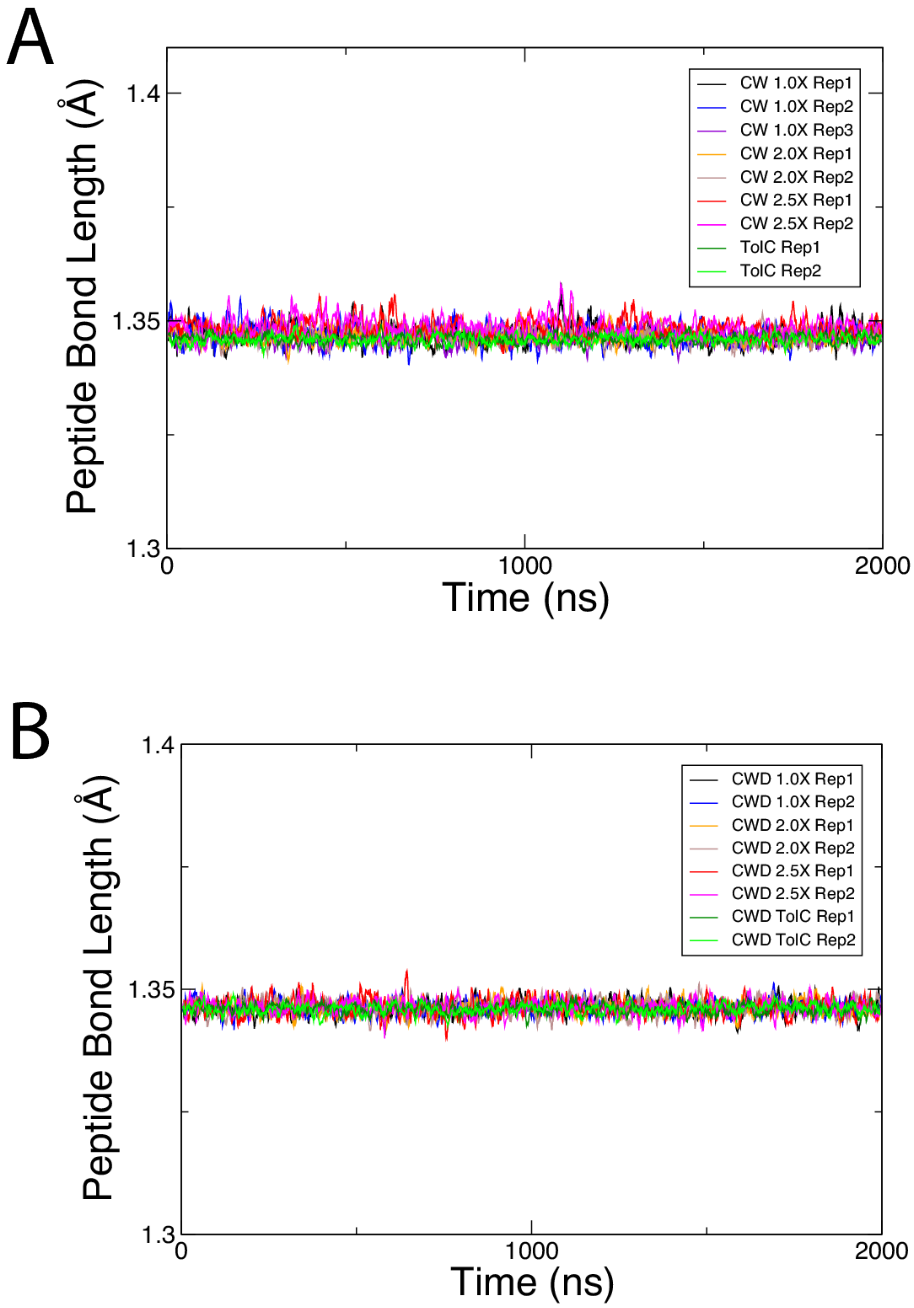

Figure S1: Average peptide bond lengths within the CW over time for all the (A) undamaged-CW and (B) damaged-CW systems. CW 1.0× systems are shown in black, blue, and violet lines. CW 2.0× systems are shown in orange and brown lines, CW 2.5× systems are shown in red and magenta lines, and TolC systems are shown in dark green and green lines.

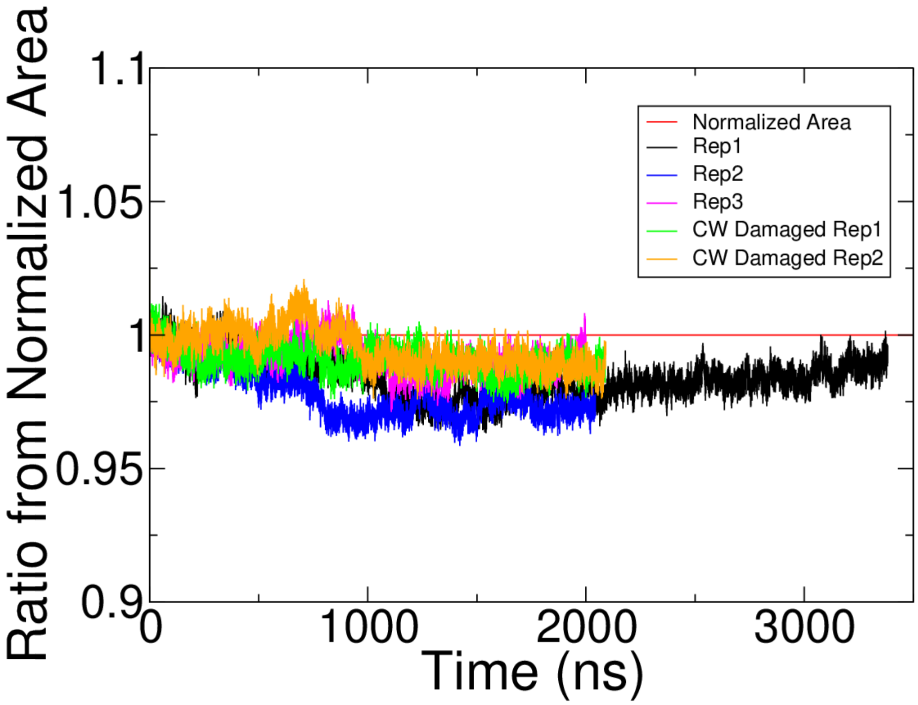

Figure S2: The ratio of membrane normal area change from the CW start area over time is shown for all the replicas of 1.0× system. The initial CW area is shown in red, and replicas 1, 2, and 3 are shown in black, blue, and magenta lines respectively. The replicas with the damaged CW are shown in green and orange, respectively.

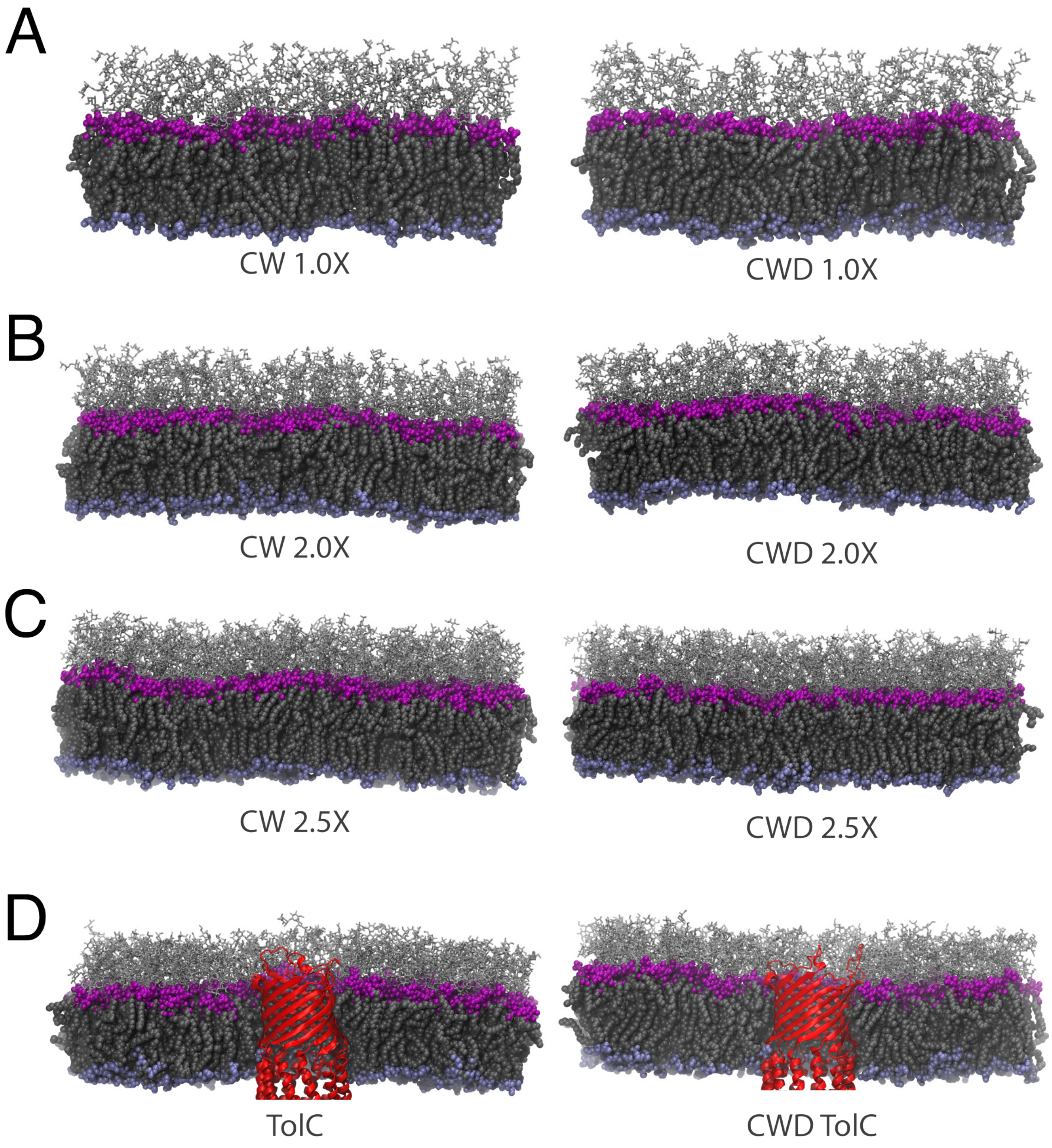

Figure S3: Comparison of the OMs for undamaged- (CW) and damaged-CW (CWD) systems. All panels show snapshots at the end of the 2-μs simulations. (A) CW 1.0× system, (B) CW 2.0× system, (C) CW 2.5× system, and (D) TolC system. The sugars in LPS are shown in silver licorice representation, and the polar heads of LPS and phospholipids are shown in magenta and ice-blue spheres, respectively. The lipid tails for both are shown in grey spheres. TolC is shown in red cartoon representation in (D).

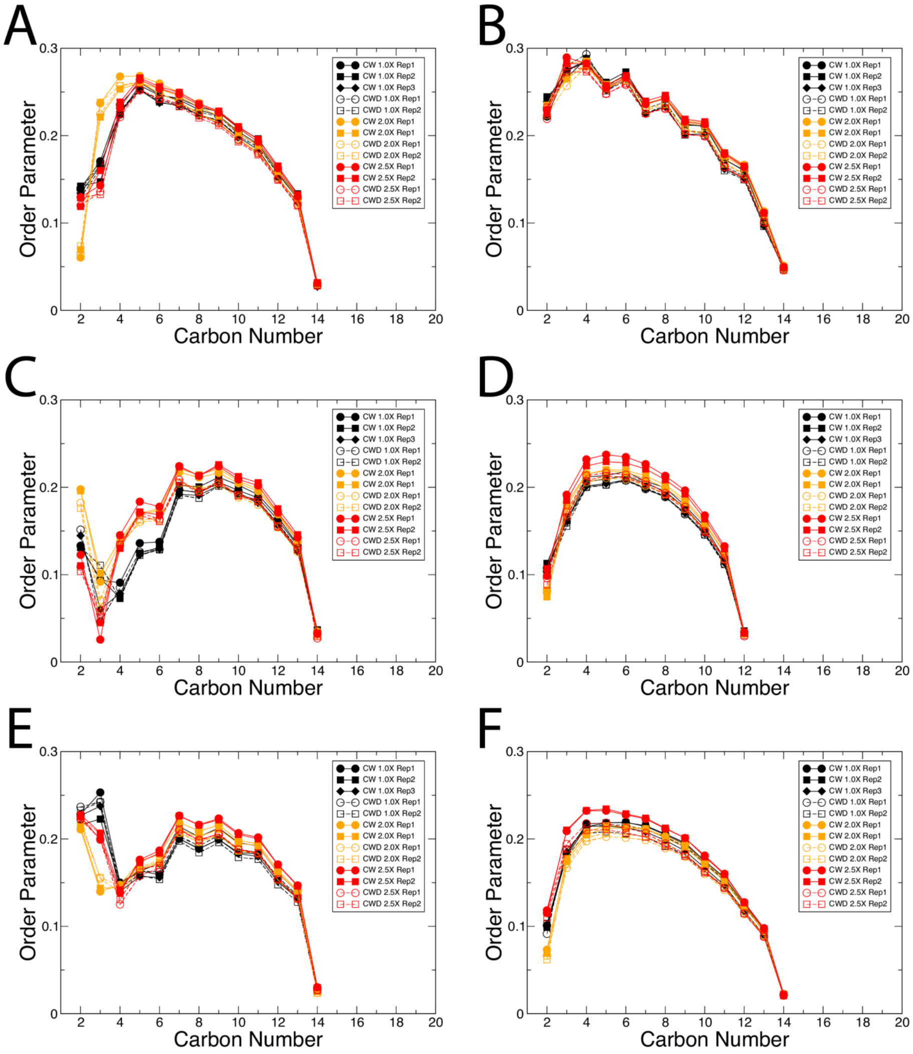

Figure S4: Order parameters of LPS from CW 1.0×, CW 2.0×, and CW 2.5× systems with undamaged or damaged CWs. Replicas 1, 2, and 3 are shown in circle, squares, and diamonds, respectively. The CW 1.0× system is shown in black, CW 2.0× in orange, and CW 2.5× system in red. The damaged-CW systems are shown in dashed lines and unfilled points. Each panel represents order parameters for one tail.

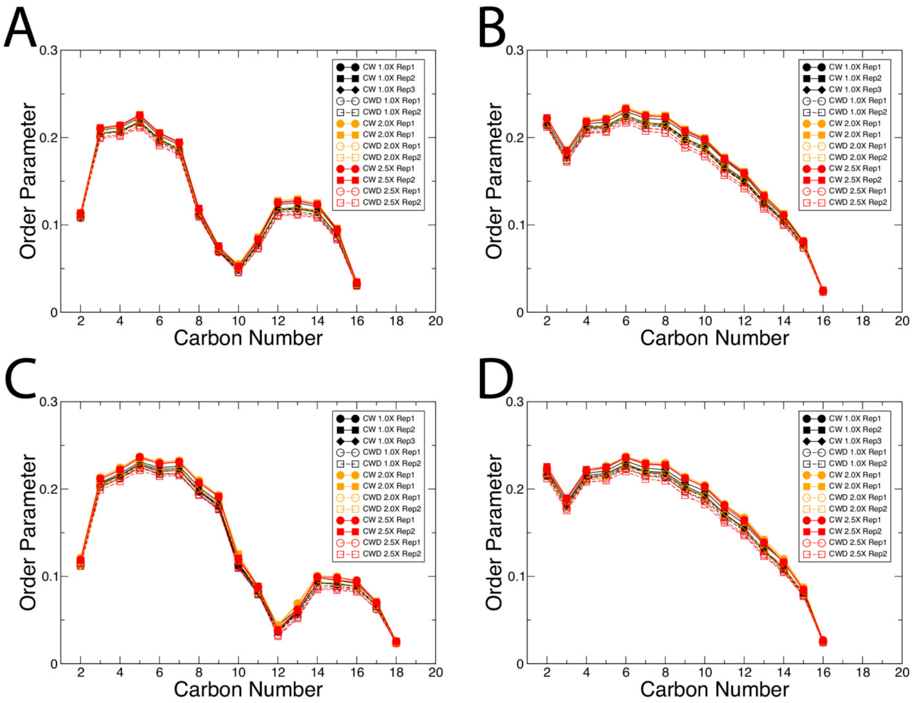

Figure S5: Order parameters of PPPE and PVPG from CW 1.0×, CW 2.0×, and CW 2.5× systems with undamaged or damaged CWs. Replicas 1, 2, and 3 are shown in circle, squares, and diamonds, respectively. The CW 1.0× system is shown in black, CW 2.0× in orange, and CW 2.5× system in red. The damaged-CW systems are shown in dashed lines and unfilled points. Each panel represents order parameters for one tail of either PPPE (panels A and B) or PVPG (panels C and D).

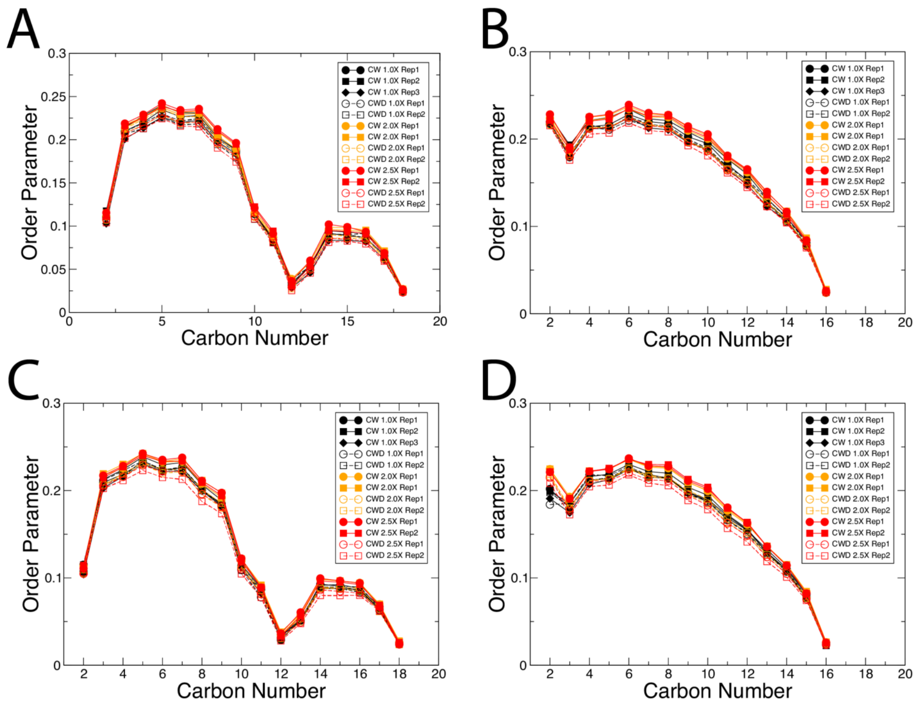

Figure S6: Order parameters of PVCL2 from CW 1.0× and CW 2.5× systems with undamaged or damaged CWs. Replicas 1, 2, and 3 are shown in circle, squares, and diamonds, respectively. The CW 1.0× system is shown in black, CW 2.0× in orange, and CW 2.5× system in red. The damaged-CW systems are shown in dashed lines and unfilled points. Each panel represents order parameters for one tail.

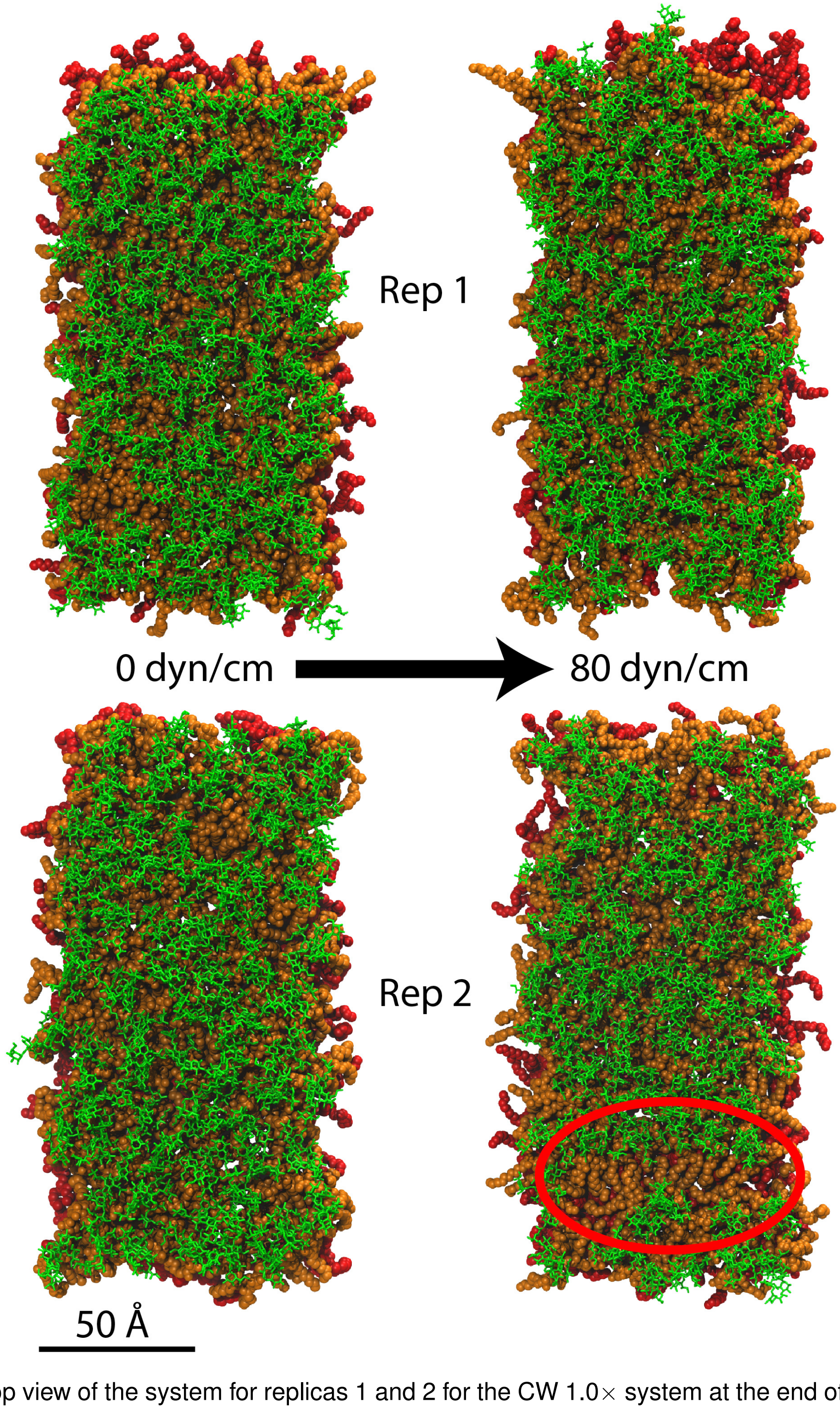

Figure S7: Top view of the system for replicas 1 and 2 for the CW 1.0× system at the end of 0 dyn/cm and 80 dyn/cm runs.

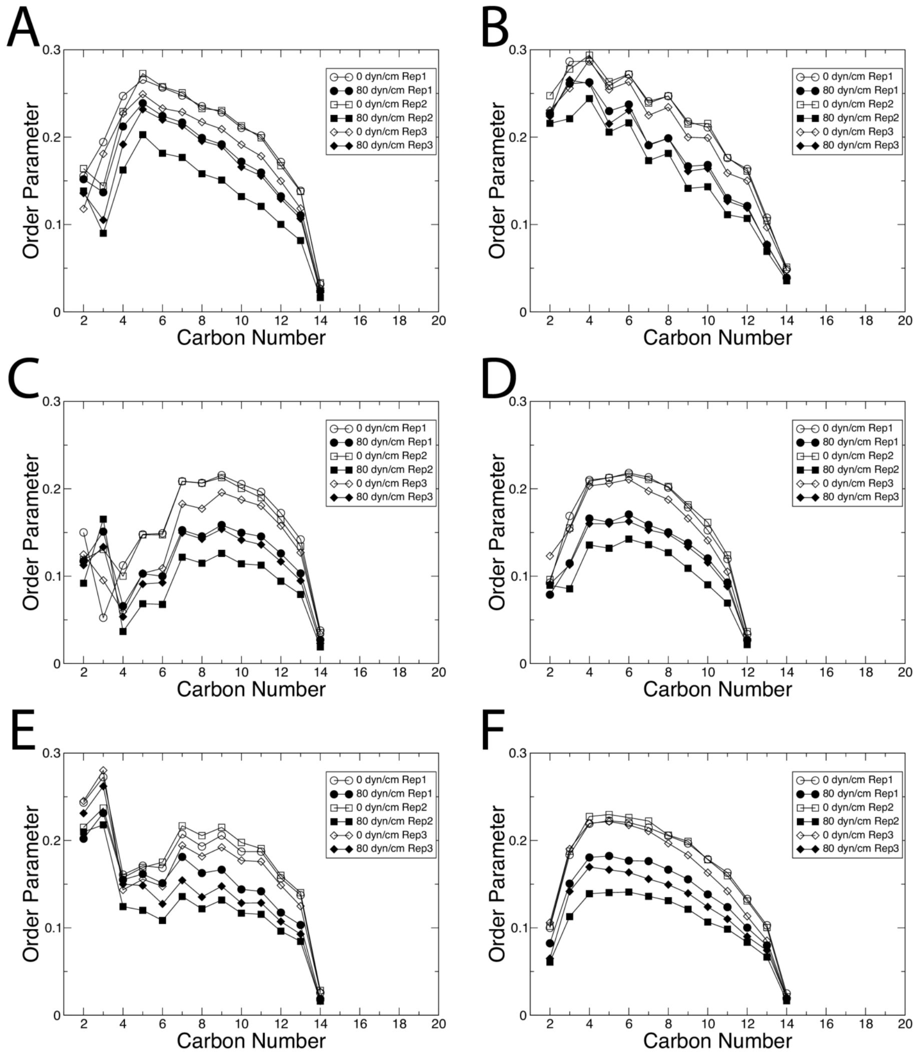

Figure S8: Order parameters of LPS from CW 1.0× systems after applying surface tensions from 0 dyn/cm to 80 dyn/cm. Replicas 1, 2, and 3 are shown in circle, squares, and diamonds, respectively. The points for 0 dyn/cm are not filled in, while those for 80 dyn/cm are filled in. Each panel represents order parameters for one tail.

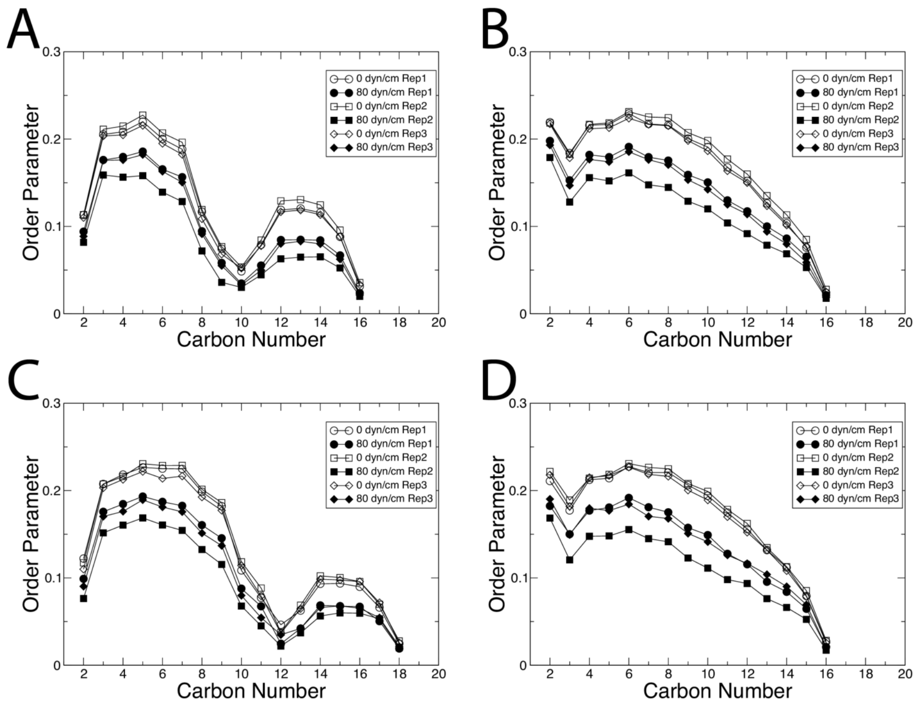

Figure S9: Order parameters of PPPE and PVPG from CW 1.0× systems after applying surface tensions from 0 dyn/cm to 80 dyn/cm. Replicas 1, 2, and 3 are shown in circle, squares, and diamonds, respectively. The points for 0 dyn/cm are not filled in, while those for 80 dyn/cm are filled in. Each panel represents order parameters for one tail of either PPPE (panels A and B) or PVPG (panels C and D).

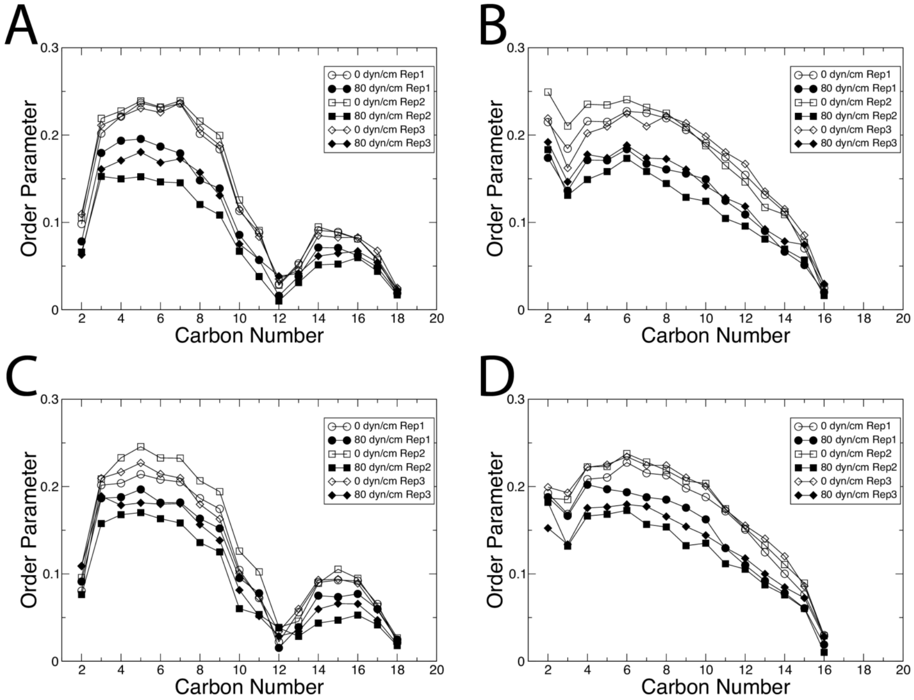

Figure S10: Order parameters of PVCL2 from CW 1.0× systems after applying surface tensions from 0 dyn/cm to 80 dyn/cm. Replicas 1, 2, and 3 are shown in circle, squares, and diamonds, respectively. The points for 0 dyn/cm are not filled in, while those for 80 dyn/cm are filled in. Each panel represents order parameters for one tail.

Table S1: Lipid compositions of OM-CW systems

CW 1.0× system

Lipid	Leaflet	Number of Lipids
E. coli LPS (Type K12)	Upper	67
PPPE	Lower	141
PVPG	Lower	37
PVCL2	Lower	8

CW 2.0× system

Lipid	Leaflet	Number of Lipids
E. coli LPS (Type K12)	Upper	134
PPPE	Lower	281
PVPG	Lower	74
PVCL2	Lower	18

CW 2.5× system

Lipid	Leaflet	Number of Lipids
E. coli LPS (Type K12)	Upper	166
PPPE	Lower	349
PVPG	Lower	91
PVCL2	Lower	20

TolC system

Lipid	Leaflet	Number of Lipids
E. coli LPS (Type K12)	Upper	186
PPPE	Lower	352
PVPG	Lower	94
PVCL2	Lower	23

Table S2: Number of ions within OM-CW systems

System	Sodium	Chloride	Magnesium	Calcium
CW 1.0×	501	359	134	201
CW 2.0×	732	549	268	402
CW 2.5×	878	693	334	501
TolC	1409	1036	804	263

Table S3: Average ratio of system area to initial normalized area over the last 200 ns for the undamaged systems

System	Replica	Average	St. Dev.
CW 1.0×	Rep1	0.980	0.004
	Rep2	0.972	0.003
	Rep3	0.991	0.004
CW 2.0×	Rep1	0.980	0.002
	Rep2	0.974	0.002
CW 2.5×	Rep1	0.953	0.002
	Rep2	0.957	0.002
TolC	Rep1	1.000	0.002
	Rep2	0.991	0.002

Table S4: Average ratio of system area to initial normalized area over the last 200 ns for the CW damaged systems

System	Replica	Average	St. Dev.
CWD 1.0×	Rep1	0.989	0.004
	Rep2	0.988	0.003
CWD 2.0×	Rep1	0.995	0.003
	Rep2	1.000	0.003
CWD 2.5×	Rep1	0.983	0.002
	Rep2	0.998	0.002
CWD TolC	Rep1	0.994	0.002
	Rep2	1.002	0.002
